# 3D vs. 2D-4 K: Performance and self-perception of laparoscopic novices in a randomized prospective teaching intervention using standard tasks and box trainers

**DOI:** 10.1007/s00423-024-03515-5

**Published:** 2024-10-30

**Authors:** Benny Kölbel, Julian Ragnitz, Kevin Schäle, Moritz Witzenhausen, Steffen Axt, Christian Beltzer

**Affiliations:** 1 Department of General, Visceral and Thoracic Surgery, German Armed Forces Hospital Ulm, Oberer Eselsberg 40, 89081 Ulm, Germany; 2 School of Medicine, HMU Health and Medical University, Olympischer Weg 1, 14471 Potsdam, Germany; 3 Department for Trauma Surgery and Orthopedics, Reconstructive and Septic Surgery, Sportstraumatology, German Armed Forces Hospital Ulm, Oberer Eselsberg, 40, 89081 Ulm, Germany; 4 Department of General, Visceral and Transplant Surgery, Tübingen University Hospital, Hoppe-Seyler-Str. 3, 72076 Tübingen, Germany

**Keywords:** Surgical training, Laparoscopy, 3D, 2D-4 K

## Abstract

**Objective:**

The use of three-dimensional (3D) laparoscopy in surgical practice and training has been an area of research and discussion. Studies have suggested that 3D vision can improve speed and precision compared to traditional two-dimensional (2D) displays, while other authors found no benefits on the learning curves of laparoscopic novices. Modern two-dimensional laparoscopy with a resolution of 3840 × 2160 pixels (2D-4 K) seems to improve laparoscopic view and helps learners orient without stereopsis. However, evidence comparing these systems for laparoscopic training is limited. Therefore, the impact of viewing mode (2D-4 K vs. 3D) on learning and task proficiency remains unclear.

**Design:**

We performed a two-hour teaching intervention on basic laparoscopic skills for novices. In this parallel group randomized study, we randomly assigned learners to 2D-4 K or 3D teaching and performed tasks of increasing difficulty and complexity using standard laparoscopy box trainers. Before the last and most challenging task, learners had to crossover to the other laparoscopy setup. Our hypothesis was that learners would be faster and more precise when using a 3D setup. The primary endpoint was task proficiency measured by speed and failure rate. Secondary outcomes were performance using the viewing mode of the other group without familiarization, self-perception, and career aspirations before and after the teaching intervention, expressed on a Likert scale.

**Setting:**

The study was performed by the Department of General, Visceral and Thoracic Surgery at the German Armed Forces Hospital Ulm, which is an academic teaching hospital of the University of Ulm.

**Participants:**

Thirty-eight laparoscopic novices, including medical students and junior residents, participated voluntarily in this teaching intervention. Group allocation was performed via the virtual coin flip method. Apparently, participants and tutors were not blinded to group assignment. No formal approval by the ethics committee was needed for this noninvasive study in compliance with the World Medical Association Declaration of Helsinki as discussed with the ethics committee of the University of Ulm.

**Results:**

Thirty-eight laparoscopy novices were randomized in the study. The 3D group (*n* = 19) was significantly faster than the 2D-4 K group (*n* = 19) (*p* = .008) in a standard box trainer model, with 134.45 ± 41.45 s vs. 174.99 ± 54.03 s for task 1 and 195.97 ± 49.78 s vs. 276.56 ± 139.20 s for task 2, and the effect was consistent throughout the learning curve. The failure rate was not significantly affected by the viewing mode. After crossover to the other laparoscopy system, precision and time were not significantly different between the groups. Learners rated the difficulty of laparoscopy lower on a Likert scale after having two hours of basic laparoscopy training. The study was funded by the hospital’s teaching budget.

**Conclusions:**

Laparoscopic novices can benefit from a 3D laparoscopy training setup. Exclusive 3D training prior to a complex task on a 2D-4 K setup does not negatively affect the learner’s performance.

## Introduction

### Background

Since the first laparoscopic cholecystectomy in 1985, which was a topic of great dispute at that time, minimally invasive surgery (MIS) has become the standard of care for a for a wide range of procedures in general surgery (e.g. MIS cholecystectomy, hernia repair, colectomy) due to its clinical advantages and patient preferences [1, 2]. In high-resource healthcare systems, the rate of MIS cholecystectomy, for example, is reported to be above 96% [3].

Technical progress has substantially improved the view and handling of laparoscopy systems since its beginning, with increasing permeation of 2D-4 K and 3D systems, as its latest iterations, into clinical practice. 3D view is also implemented in modern robotic systems used in general surgery.

## Rationale and knowledge gap

As MIS has become the standard of care for both routine and complex general surgery procedures, the question arises of how learners are best taught in this skill. Since 2D laparoscopy has been the standard of care for decades, surgeons have learned to compensate for the lack of depth perception by experience and rely on secondary visual cues such as light and shadow, the relative size of organs, and the movement of instruments [4–8]. While the hypothetical benefits of 3D laparoscopy are obvious, surgeons report side effects such as vertigo, eye strain and headaches while performing 3D laparoscopic surgery [9]. Multiple studies have been conducted to compare the performance of learners since the first 3D laparoscopy systems became available in the 1990s. The majority of these studies used a box trainer setup, and the results tend to favor 3D regarding speed and precision [9–13], while other recent studies failed to reproduce that effect [7].

One cause of bias might be that most of those publications compared the latest 3D systems with standard 2D setups instead of 2D-4 K. Evidence using 2D-4 K setups is limited [8, 14–16]. Another question is whether skills acquired on 3D systems can be translated into clinical practice where 2D systems are still predominantly used.

## Objective

Our objective was to replicate prior findings of laparoscopic performance and learning curves of laparoscopy novices, including speed and precision, using state-of-the-art high-quality 2D-4 K and 3D laparoscopy systems. Additionally, we examined performance after crossover to the viewing mode in which the learner was not familiarized before, reflecting the clinical reality that this system might later not be available to them.

## Material and methods

According to the CONSORT Statement and checklist we drafted a randomized parallel group teaching intervention on laparoscopic novices performing basic laparoscopic tasks allocated with a ratio of 1:1 to 3D and 2D-4 K laparoscopy setups on standard box trainers, which are used in the well-established laparoscopy course at the University of Tübingen [13]. Two different laparoscopy setups were used (Olympus Surgical Technologies Europe, Hamburg, Deutschland). The 2D system was equipped with an OTV-S400 processor, a ULTRA telescope 4 K camera head and a 30° optic to offer the latest 2D technology at 4 K resolution. The 3D setup was equipped with an OTV-S300 processor, a 30° ENDOEYE and passive polarized 3D glasses or 3D clips on glasses. Both systems were connected to 32″ monitors with 3840 × 2150 pixels for the 2D-4 K setup and 1920 × 1080 pixels for the 3D setup (Sony Olympus Medical Solutions Inc., Tokyo, Japan) at the height of the participants’ eye level and at a distance of 1.5 m to ensure optimal ergonomics and comparability.

**Fig. 1:**
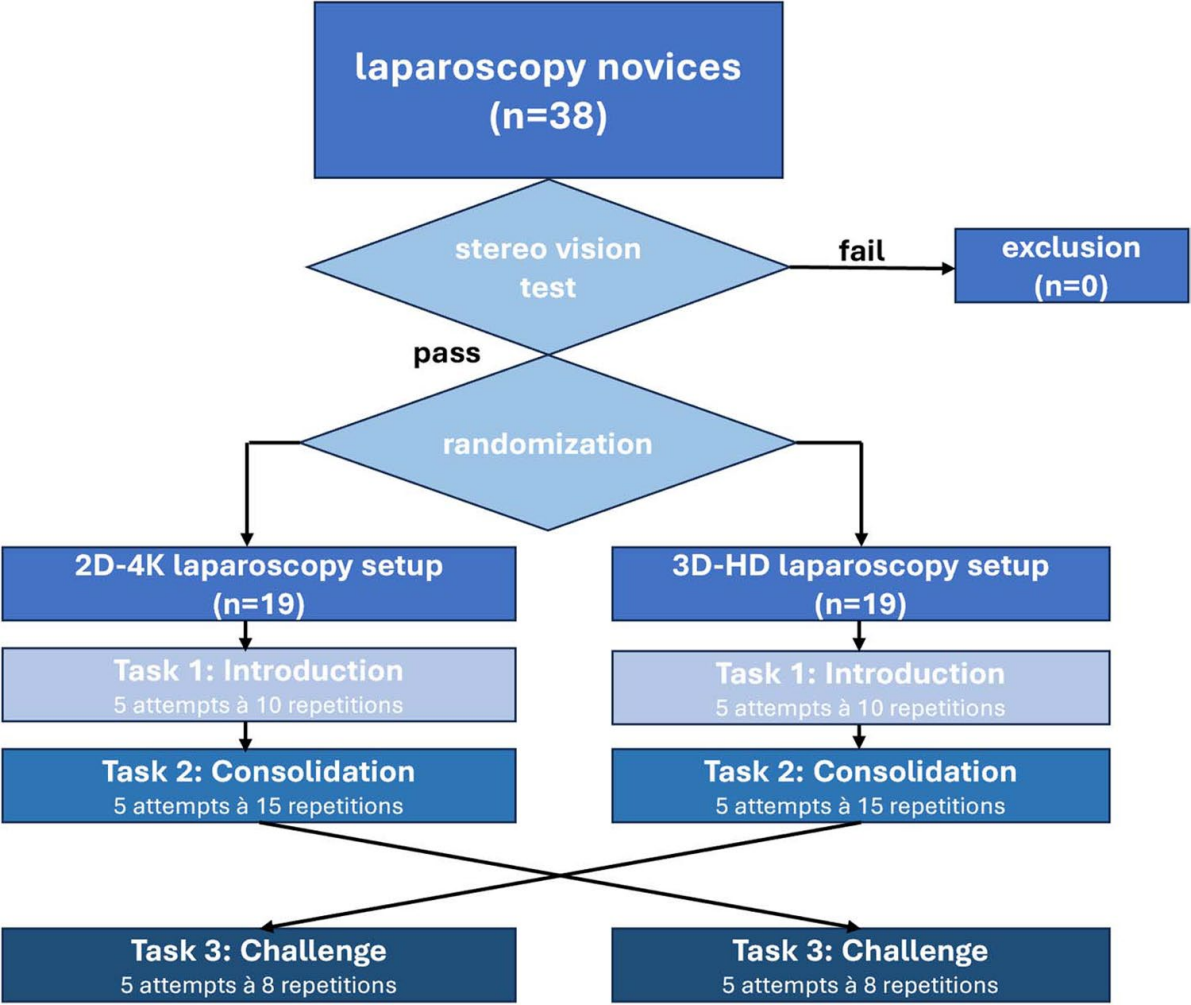
Flow chart of the 3D vs. 2D-4 K laparoscopy training for novice study, procedures and tasks

Considering a learner-to-tutor ratio of 2:1 and data suggesting the plateauing of learning curves at approximately 10 repetitions, we designed a 2-h basic laparoscopy training (refer to Fig. 1) [4, 17].

**Fig. 2:**
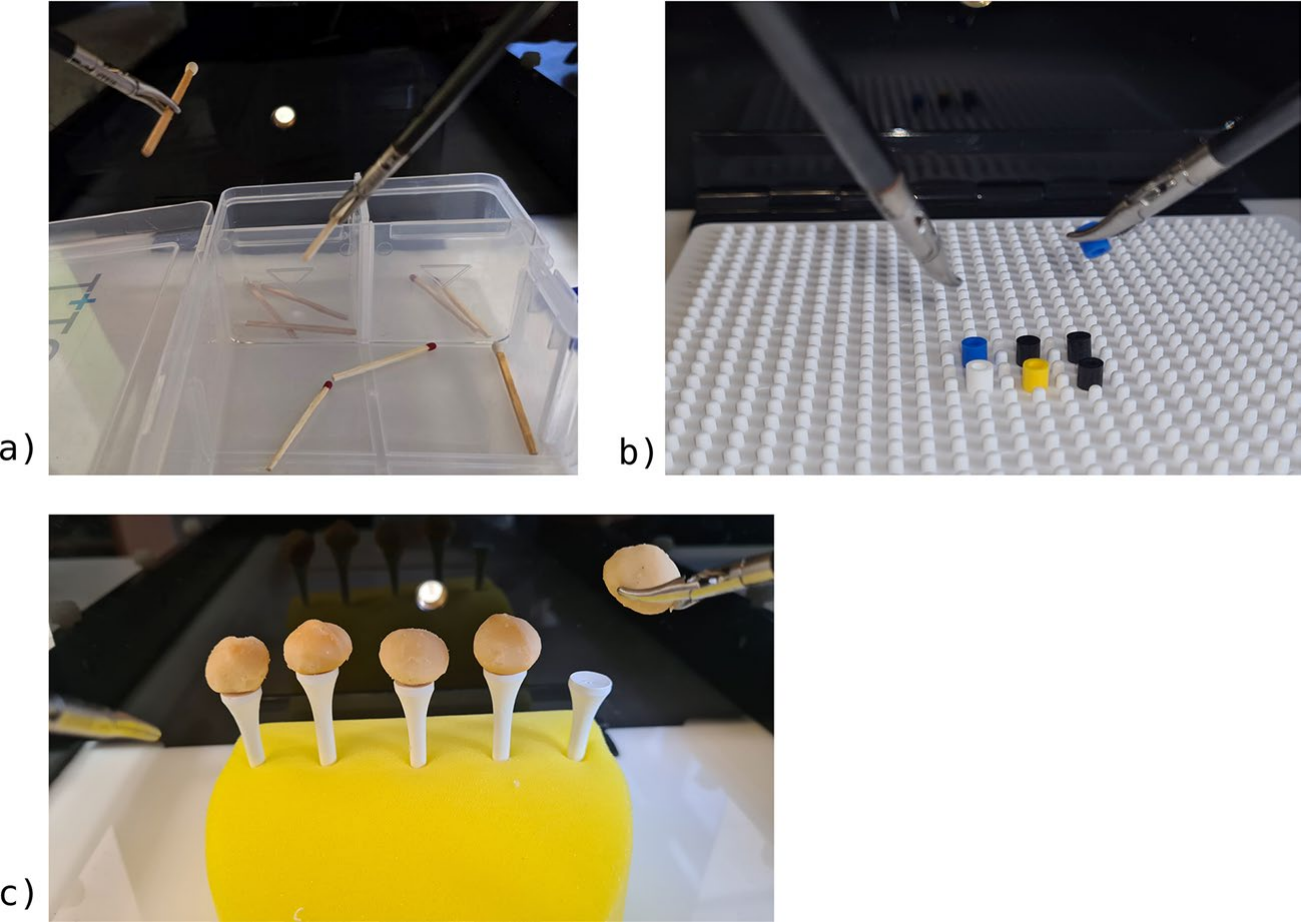
Laparoscopy training setup of the 3D vs. 2D-4 K laparoscopy training for novice study: Task 1 = 5 attempts at 10 repetitions of transfer and sorting of matches into boxes; Task 2 = 5 attempts at 15 repetitions of peg transfer; Task 3 = 5 attempts at 8 repetitions of positioning nuts on golf tees; **a**) Task 1; **b**) Task 2, **c**) Task 3

The study was performed at the German Armed Forces Hospital Ulm, which is an academic teaching hospital of the University of Ulm. The course started with a theoretical and technical introduction to the laparoscopy setup before the participants were asked for consent to participate in the study. All participants were medical students or junior residents within their first two postgraduate years (PGY-1/PGY-2) of general surgery, orthopedic surgery, or vascular surgery without prior laparoscopic experience. The participants were surveyed with a questionnaire on gender, video gaming experience, career aspirations in general surgery pre- and post-intervention, and how difficult they were to rate the skill of laparoscopy pre- and post-intervention on a Likert scale, with 0 being the lowest and 10 being the highest. The light in the training room was set to be constant over the 4-day study period for comparability. After consent was given, participants were checked for stereopsis using a Lang stereo test (Hübel, Olfen, Germany) and randomly assigned to the 3D or 2D-4 K laparoscopy system using a virtual coin-flip application (Random.org, Dublin, Ireland) by the chief investigator. They had to perform tasks of increasing difficulty and complexity. The tasks were selected to be rapidly repeatable to measure learning curves and have nothing in common with other surgical techniques (e.g. suturing or cutting) to minimize bias due to different levels of pre-existing experience in open surgery. The tasks are mainly focused on grasping of objects to reflect laparoscopic vision, without being manually overly challenging, as this would have been difficult to train in a 2-h teaching intervention. Every task consisted of 5 attempts. For task 1, every attempt contains 10 repetitions of grasping, transfer and sorting of matches into boxes; for task 2 every attempt contains 15 repetitions of peg transfer; and for task 3 every attempt contains at 8 repetitions of positioning nuts on golf tees after crossing over to the nonfamiliar laparoscopy setup. For this exercise only the successes were counted, so 8 out of 8 was the maximum score. A repetition was considered a success if the nut was laparoscopically grasped from the bottom of the box trainer and placed on the golf tee without falling off. (refer to Fig. 2) The tasks were shown by a tutor and criteria of failed attempts were outlined (e.g. match was incorrectly sorted, match/peg was dropped instead of placed). The learners had no possibility of familiarization before the measurements began in order to analyze the full learning curve.

Documentation of failed attempts and timing were performed by the supervising tutor. Tutors were PGY-4 and above surgical residents who underwent standardized laparoscopic training in well-established courses prior to the study and performed laparoscopic surgery on a regular basis. Data was then transferred to a spreadsheet (Microsoft Excel, version 365, Redmond, USA). The primary endpoint was task proficiency measured by the speed and failure rate of the different tasks. Secondary outcomes were performance using the viewing mode of the other group without familiarization, self-perception, and career aspirations before and after the teaching intervention, expressed on a Likert scale. Apparently, participants and tutors were not blinded to group assignment.

Based on our prior internal experience, we defined a 15% reduction in time in the 3D group vs. the 2D-4 K group as a meaningful improvement. With an estimated standard deviation of 20% of the overall procedural time, a group size of 19 test persons per study arm was required to achieve a power of 80% by assuming an alpha level of α = 0.05.

Statistical analysis was performed using SPSS statistical software (version 28; IBM Inc., Armonk, NY, USA). The data are presented as the means with standard deviations (SDs). Group comparisons were performed with a chi-squared test for categorical data, the Mann–Whitney U test for continuous variables, and the Wilcoxon signed-rank test for dependent samples. For repetitive testing, we used repeated-measures ANOVA. The Mauchly test was performed to test for sphericity. Violations of sphericity were corrected using the Greenhouse–Geisser adjustment for ε < 0.75 and the Huynh–Feldt adjustment for ε > 0.75. The level of significance was defined as *p* = 0.05.

For this noninvasive study, no formal approval by the ethics committee was needed in compliance with the World Medical Association Declaration of Helsinki, as discussed with the ethics committee of the University of Ulm on 13th January 2021.

## Results

### Participants characteristics

Age ranged from 22 to 43 years, with 25.8 ± 3.7 years in the 3D group and 28.0 ± 4.5 years in the 2D-4 K group. A total of 9/19 participants in the 3D group were postgraduate junior residents of surgery vs. 14/19 participants in the 2D-4 K group. In both arms, 7/19 participants had more than one hour of video gaming experience per week. None of the findings were statistically significant (refer to Table 1).

**Table 1:** Demographics and features of participants of the 3D vs. 2D-4 K laparoscopy training for novice study

*Study participants*			
Parameter	3D (*n* = 19)	2D (*n* = 19)	*p*-value
Age [mean ± SD]	25.8 ± 3.7	28.0 ± 4.5	.11*
Postgraduate status	9	14	.10**
Weekly videogaming			.73*
none	12	12	
1–4 h	5	5	
more than 4 h	2	2	

*SD*, standard deviation; *3D*, three-dimensional laparoscopic viewing system; *2D*, two-dimensional laparoscopic viewing system. *Mann‒Whitney U test, **χ2 test

### Primary outcome

All participants received the assigned teaching intervention and were included in the data analysis. The time to completion of the first attempt of task 1 was 182.5 ± 57.8 s in 3D vs. 231.3 ± 79.8 s in 2D, while the time to completion of the last attempt of task 1 was 107.2 ± 35.6 s in 3D vs. 142.7 ± 44.8 s in 2D. The time to completion of the first attempt of task 2 was 240.7 ± 61.0 s in 3D vs. 356.4 ± 213.2 s in 2D, while the time to completion of the last attempt of task 2 was 171.1 ± 50.9 s in 3D vs. 236.9 ± 92.0 s in 2D. The 3D and 2D intervention groups differed significantly regarding time for tasks 1 and 2, F (1, 36) = 7.94, *p* = 0.008, partial η^2^ = 0.18.

The intervention groups did not differ significantly regarding failure rates for tasks 1 and 2, F (1, 36) = 3,32, *p* = 0.077, partial η^2^ = 0.09.

Repeated measures ANOVA with a Greenhouse‒Geisser correction revealed that the mean times of all participants decreased significantly in tasks 1 and 2 with ongoing training, with F_t_task1_(2,76, 99.50) = 36.98, *p* < 0.001, partial η^2^ = 0.51 and F_t_task2_(2,13, 76.49) = 21.46, *p* < 0.001, partial η^2^ = 0.37.

**Fig. 3:**
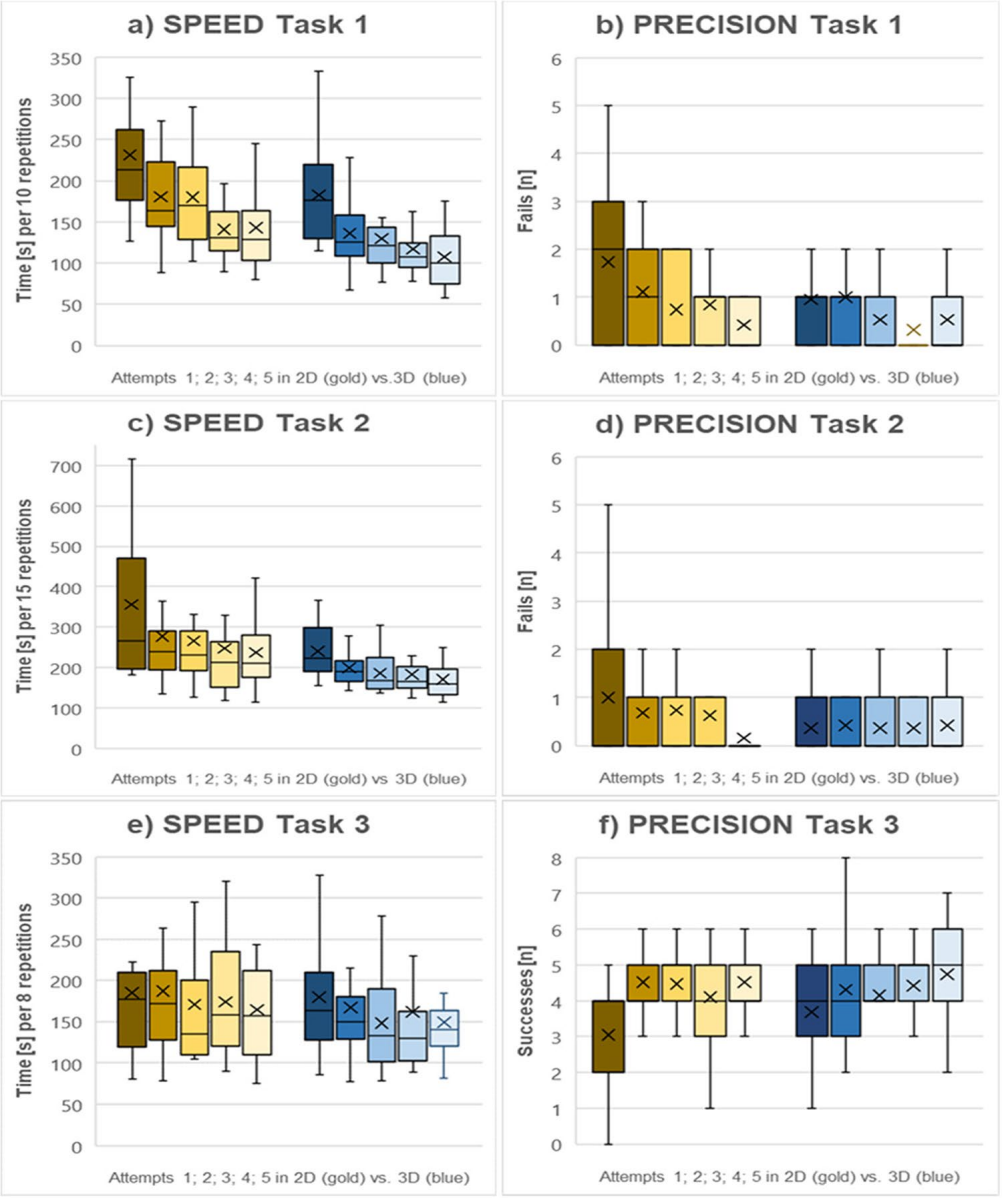
Boxplots of speed and precision in the 3D vs. 2D-4 K laparoscopy training for novice study. Horizontal bands indicate quartiles, crosses indicate means, horizontal bands within the Tuskey box reflect the median, and whisker lines indicate the 2.5th and 97.5th percentiles

The failure rates of all participants decreased significantly in task 1, with F_f_task1_(3.49, 125.62) = 5.87, *p* < 0.001, partial η^2^ = 0.14, but not significantly in task 2, with F_f_task2_(3.78, 136.10) = 1.31, *p* = 0.27, partial η^2^ = 0.04 (refer to Table 2 and Fig. 3).

**Table 2:** Primary outcome of the 3D vs. 2D-4 K laparoscopy training for novice study

*Primary outcomes*			
Parameter	3D (*n* = 19)	2D (*n* = 19)	*p*-value*
Speed Task 1 [s; mean ± SD]			.008*
1st attempt	182.5 ± 57.8	231.3 ± 79.8	
2nd attempt	135.8 ± 36.9	180.3 ± 51.6	
3rd attempt	129.8 ± 41.1	179.9 ± 55.3	
4th attempt	116.8 ± 35.8	140.7 ± 38.7	
5th attempt	107.2 ± 35.6	142.7 ± 44.8	
Speed Task 2 [s; mean ± SD]			
1st attempt	240.7 ± 61.0	356.4 ± 213.2	
2nd attempt	199.4 ± 42.1	276.7 ± 141.9	
3rd attempt	186.2 ± 46.4	265.5 ± 119.7	
4th attempt	182.4 ± 48.5	247.3 ± 129.2	
5th attempt	171.1 ± 50.9	236.9 ± 92.0	

*SD*, standard deviation; *3D*, three-dimensional laparoscopic viewing system; *2D* two-dimensional laparoscopic viewing system. *repeated-measures ANOVA

## Secondary outcomes

After crossover to the other viewing mode, the 3D group had 4.3/8 ± 1.3 successes vs. 4.1/8 ± 1.3 successes in the 2D-4 K group. The time for task 3 was 161.3 ± 67.5 s in 3D vs. 176.5 ± 79.0 s in 2D-4 K.

The intervention groups did not differ significantly regarding time for task 3, with F_t_task_3_(1, 36) = 0.48, *p* = 0.49, partial η^2^ = 0.01, or success rate, with F_s_task_3_(1, 36) = 1.11, *p* = 0.30, partial η^2^ = 0.03.

Learners rated the perceived difficulty of laparoscopy significantly lower after the teaching intervention on a 0–10 Likert scale (6.42 ± 1.74 vs. 4.87 ± 1.17, *p* =  < 0.001).

The participants rated the likelihood of pursuing a career in general surgery significantly more after the teaching intervention on a 0–10 Likert scale (4.55 ± 2.72 vs. 4.87 ± 2.90, *p* = 0.02).

The performance times for tasks 1 and 2 in laparoscopy novices with > 1 h of experience in videogaming per week were not significantly different (refer to Table 3).

**Table 3:** Secondary outcomes of the 3D vs. 2D-4 K laparoscopy training for novice study

*Secondary outcomes*				
Parameter	3D (*n* = 19)	2D (*n* = 19)	Overall (*n* = 38)	*p*-value
Performance after crossover to non-familiar system				
Successes [n; mean ± SD]	4.3/8 ± 1.3	4.1/8 ± 1.3		.30*
Time [s; mean ± SD]	161.3 ± 67.5	176.5 ± 79.0		.49*
Self-perception of difficulty of laparoscopy				
Pre teaching [0–10, mean]	6.63	6.21	6.42 ± 1.74	.39**
Post teaching [0–10, mean]	5.00	4.74	4.87 ± 1.17	.51**
Intervention effect [Δ; mean ± SD]	-1.63 ± 1.42	-1.47 ± 1.67	-1.55 ± 1.55	< .001***
Career aspiration in general surgery				
Pre teaching [0–10, mean]	4.79	4.32	4.55 ± 2.72	.62**
Post teaching [0–10, mean]	5.11	4.63	4.87 ± 2.90	.60**
Intervention effect [Δ; mean ± SD]	0.32 ± 0.65	0.32 ± 0.80	0.32 ± 0.73	.02***
Gamers				
Participant has > 1 h experience in videogaming per week, Time for all attempts of Task 1/2 [s; mean ± SD]			[*n* = 14]	
			172.52 ± 52.3	
Participant has < 1 h experience in videogaming per week, Time for all attempts of Task 1/2 [s; mean ± SD]			[*n* = 24]	12*
			201.31 ± 56.8	

*SD*, standard deviation; *3D*, three-dimensional laparoscopic viewing system; *2D*, two-dimensional laparoscopic viewing system. On the Likert scale, 0 was the lowest, and 10 was the highest. *repeated-measures ANOVA, **Mann‒Whitney U test; ***Wilcoxon signed rank sum test

## Discussion

### Key findings

Laparoscopy novices were significantly faster when using a 3D laparoscopy setup in a standard box trainer model, and the effect was consistent throughout the learning curve. The failure rate was not significantly affected by the viewing mode. After crossover to the nonfamiliar system, precision and time were not significantly different between the groups and learners rated the difficulty of laparoscopy lower after having 2 h of basic laparoscopy training on a Likert scale. We hypothesize that learning on a 3D system, does not negatively impact performance using 2D systems.

## Strengths and limitations

A strength of this study is the utilization of two state-of-the-art laparoscopic setups to compare 2D-4 K and 3D views regarding the learning and performance of laparoscopic novices, while the different resolutions of the two setups might be a source of bias. We deliberately included a transfer task with increased manual difficulty after crossover to the nonfamiliar laparoscopic viewing modality to gain insight into the transferability of laparoscopic skills acquired on 3D systems, when being forced to work in 2D as those systems will remain the clinical standard and reality in most hospitals for the next few years. While showing no significant difference in time and precision after crossover, our data are based on laparoscopic novices only and cannot necessarily be translated to experienced surgeons, although there is evidence that experienced laparoscopy providers may also benefit from 3D vision [18]. Additionally, the generalizability of this finding must be questioned, as task 3 was more challenging in terms of fine motor control compared to tasks 1 and 2 with more sweeping movements that rely on depth perception. We did test for stereopsis using the semi-quantitative Lang test. As Gietzelt et al. showed that the level of stereo-vision is of great importance for the performance in 3D laparoscopic tasks and more advanced assessments of stereo-vision might have been beneficial to control for this possible source of bias [19].

Another limitation of this study is the simulator-based design. However, how our results translate into learning curves for standard MIS procedures for laparoscopic learners remains unclear. The sample size was calculated for the performance time of tasks 1 and 2 and not for precision or the transfer task 3. A larger sample size might have shown differences regarding the failure rates.

Transferability into clinical practice is also limited as the tasks were picked to be feasible for novices and reflect vision and depth perception as this was the primary objective of this study.

## Comparison with similar research

Multiple studies and meta-analyses have shown that 3D laparoscopy training seems to be superior to 2D laparoscopy training for learners [4, 9–12, 20] although recent data have failed to replicate that effect in a very similar study design [7].

Although studies on 3D vs. 2D-4 K remain limited, our study replicated the findings of Thomaschweski et al. using very similar laparoscopy setups. They found that 3D is beneficial in basic tasks with wide-range movements, but 2D-4 K is as good as 3D if the tasks are within confined spaces or require particular precision, as seen in task 3 in our study [15].

The studies of Sørensen et al. and Kunert et al. support our finding that the learning effects of 3D training are transferable to 2D systems and that laparoscopy performance might predominantly be affected by the system the operator is actually using and less by the system he trained at [10, 20]. The influence of the viewing mode is also discussed with robotic systems in general surgery. Although there is sound rationale and some data on the superiority of robotic systems compared to 3D and 2D laparoscopy systems, there is the possibility for bias due to the variety of robotic platforms and the substantial preexisting experience of the participants in robotic surgery studies [21, 22].

## Explanations of findings

The hypothetical benefits of 3D vision on depth perception and procedural speed are self-evident and were generally replicated in this study, but the effect of 3D vision was not significant in transfer task 3. As 2D-4 K offers four times the resolution of HD it can give more monocular cues of depth perception especially in a narrow surgical field like in task 3 [8, 16].

We subsequently surveyed the participants’ career aspirations and perceptions of the difficulty of laparoscopy. Although not surprising, it is reassuring that learners rated laparoscopy easier after basic teaching, showing that structured teaching helps learners overcome the challenges of this psychomotor skill [4, 23].

Despite being elective course students or even surgical residents within the first year of training, participants gave only an intermediate rating for the likelihood of pursuing a career in general surgery, which slightly improved with the teaching intervention. This might be explained by the current challenges of surgical training and practice [24].

## Implications and actions needed

Our study shows that considerable training effects are possible within a brief teaching intervention using the concept of deliberate practice on both 3D and 2D-4 K laparoscopy setups. Early psychomotor skill training is of great importance, as laparoscopic surgery is more challenging to learn than open surgery due to many factors like the loss of stereoscopic vision and the fulcrum effect [23, 25, 26].

Wherever possible, 3D systems should be considered for the replacement of laparoscopy systems that have reached their end-of-lifetime. If the effects of procedural speed from the box trainer model translate into clinical practice, even earlier replacement might be economically reasonable due to reduced operating times.

## Conclusions

Laparoscopic novices can benefit from a 3D laparoscopy training setup. Having exclusively 3D training prior to a difficult task on a 2D setup did not negatively affect the learner’s performance in this setup. Deliberate practice on standard box trainers is an essential part of structured laparoscopic curricula to improve learners´ performance in this challenging psychomotor skill.

## Data Availability

The datasets generated and analysed in this study are available from the corresponding author upon reasonable request.

## Abbreviations

2D: Two-dimensional laparoscopic viewing
2D-4K: Two-dimensional laparoscopic viewing with a resolution of 3840 × 2160 pixels
3D: Three-dimensional laparoscopic viewing system
CONSORT: Consolidated Standards of Reporting Trials
MIS: Minimally invasive surgery
PGY: Postgraduate year
SD: Standard deviation
